# Continuous combined oral contraceptive use versus vitamin E in the treatment of menstrual migraine: rationale and protocol of a randomized controlled trial (WHAT!)

**DOI:** 10.1186/s13063-024-07955-8

**Published:** 2024-02-15

**Authors:** Britt W. H. van der Arend, Daphne S. van Casteren, Iris E. Verhagen, Antoinette MaassenVanDenBrink, Gisela M. Terwindt

**Affiliations:** 1 Department of Neurology, Leiden University Medical Center, PO Box 9600, Leiden, 2300 RC The Netherlands; 2 Division of Vascular Medicine and Pharmacology, Department of Internal Medicine, Erasmus Medical Center, Rotterdam, The Netherlands

**Keywords:** Migraine, Headache, Hormones, Treatment, Combined oral contraceptive pill

## Abstract

**Background:**

Currently, there is no evidence-based hormonal treatment for migraine in women. Several small studies suggest a beneficial effect of combined oral contraceptives, but no large randomized controlled trial has been performed. As proof of efficacy is lacking and usage may be accompanied by potentially severe side effects, there is a great need for clarity on this topic.

**Methods:**

Women with menstrual migraine (*n* = 180) are randomly assigned (1:1) to ethinylestradiol/levonorgestrel 30/150 μg or vitamin E 400 IU. Participants start with a baseline period of 4 weeks, which is followed by a 12-week treatment period. During the study period, a E-headache diary will be used, which is time-locked and includes an automated algorithm differentiating headache and migraine days.

**Results:**

The primary outcome will be change in monthly migraine days (MMD) from baseline (weeks − 4 to 0) to the last 4 weeks of treatment (weeks 9 to 12). Secondary outcomes will be change in monthly headache days (MHD) and 50% responder rates of MMD and MHD.

**Conclusions:**

The WHAT! trial aims to investigate effectivity and safety of continuous combined oral contraceptive treatment for menstrual migraine. Immediate implementation of results in clinical practice is possible.

**Trial registration:**

Clinical trials.gov NCT04007874. Registered 28 June 2019.

## Background

Sex hormones have long been known to play a prominent role in the pathophysiology of migraine. Menstruation is an important factor increasing the susceptibility for an upcoming attack, with the highest risk in the period of 2 days before the menstrual period until the first 3 days of bleeding [1]. Before the onset of menarche, the prevalence of migraine is equal between prepubertal boys and girls, but subsequently the balance shifts towards an increased prevalence of migraine in women, with a peak prevalence between 30 and 40 years of age [2]. Hormonal fluctuations during menopausal transition are also associated with increased susceptibility for migraine [3]. Furthermore, the majority of women with migraine without aura report improvement of their migraine attacks during pregnancy and breastfeeding [4, 5]. Migraine with aura can also improve during pregnancy but more often remains the same or worsens [6, 7].

Despite the well-documented relationship between sex hormones and migraine in women, the underlying mechanisms through which hormonal factors influence migraine susceptibility remain poorly understood. It is hypothesized that perimenstrual migraine might be due to the sudden drop in estradiol prior to menses [8–10]. However, a similar decrease in circulating estradiol occurs at ovulation, but this decline does not seem to be consistently related to the provocation of migraine attacks. This may be due to the preventive properties of increasing progesterone levels during ovulation or its derivate allopregnanolone [11, 12]. With this estrogen withdrawal theory in mind, some small studies suggest that combined oral contraceptives might have beneficial effects in migraine [13–15]. A small double-blind placebo-controlled crossover study found a 22% reduction in migraine frequency after daily administration of estradiol (1.5 mg) [16]. However, a 40% increase in migraine frequency was observed 5 days after cessation of the intervention. It is therefore suggested that a migraine attack can be delayed by preventing the estrogen withdrawal prior to menstruation.

Combined oral contraceptives (COC) are the most prescribed type of combined hormonal contraception (CHC). They are primarily composed of an estrogen (typically ethinylestradiol) and progestin to prevent pregnancy in women of childbearing age. The composition of COC includes a wide range of hormones, doses, and regimes. The 21/7 regimen, in which 21 days of pill administration is followed by a 7-day hormone-free interval (HFI), is the most common. As the drop in estrogen levels prior to menstruation is suggested to provoke migraine attacks, studies have been investigating the effect of a shorter or absent HFI and of estrogen supplementation during the HFI on the occurrence of menstrual migraine [10, 17]. Shortening the HFI might also lead to a reduction in migraine intensity and frequency [14, 18]. However, worsening of symptoms was also observed in some cases. Extended regimens (without HFI) were found to be more effective than the traditional 21/7 regimen in terms of reducing daily headache days and medication days [19, 20].

In a small study, a beneficial effect of vitamin E was found with respect to pain severity and functional disability [21]. Its effect was suggested to be mediated by a reduction of prostaglandin production in the endometrium. The study, notwithstanding, had methodological shortcomings, such as a short duration of treatment solely during the perimenstrual period and a lack of clearly defined outcome definitions.

In clinical practice, combined oral contraceptive pills are frequently prescribed for migraine in women. However, proof of efficacy is lacking and usage may be accompanied by side effects, with an increased relative risk for stroke in young women with migraine [22, 23]. Hence, there is a strong need for clarity on this topic. We hypothesize that continuous daily use of a combined oral contraceptive pill will be an effective and well-tolerated preventive treatment for menstrual migraine.

## Methods

**Fig. 1:**
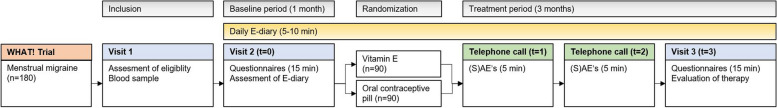
Flowchart study design

**Fig. 2:**
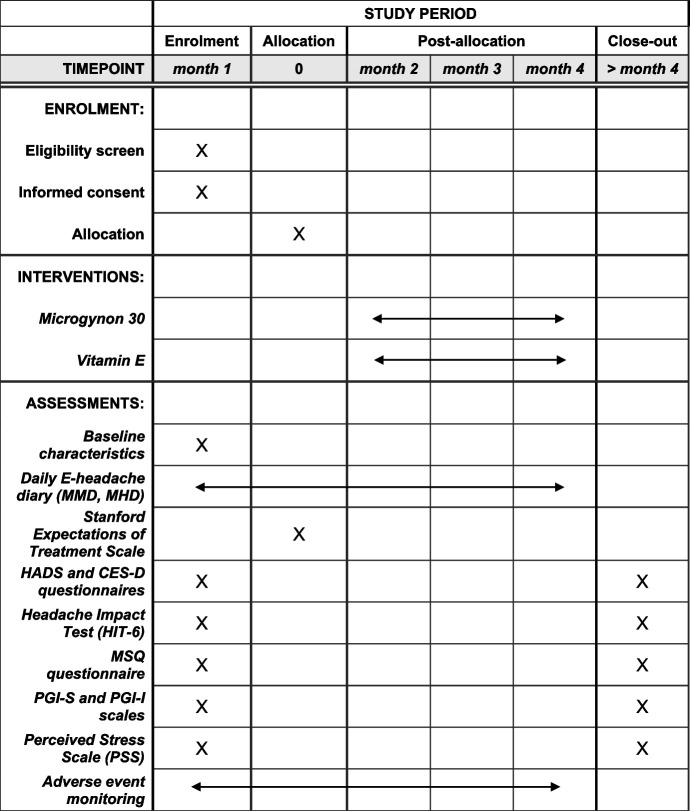
SPIRIT figure for the schedule of enrolment, interventions, and assessments

The primary objective of this randomized controlled trial is to investigate the efficacy of continuous daily use of ethinylestradiol/levonorgestrel (30/150 μg/day) compared to vitamin E (400 IU/day) in treating menstrual migraine. This study follows the latest protocol, dated August 1, 2022, version 5.0. This trial employs an open-label randomized controlled design due to ethical and practical considerations regarding the use of a placebo-controlled study with a combined oral contraceptive pill. However, a control group with an alternative treatment, vitamin E (400 IU/day, soft gel capsules), was included to account for potential confounding biases such as treatment expectations and placebo and nocebo effects. Our patient focus group panel was highly in favor of using vitamin E as a comparator treatment for hormonal intervention. Given the nature of the interventions, participants, investigators, and study personnel are aware of the treatment assignments. Blinding is not feasible in this trial due to the distinct characteristics of the interventions and the practical challenges associated with a placebo-controlled study involving a combined oral contraceptive pill. The study design is visualized in Fig. 1. All endpoints are defined as the mean change from baseline (week − 4 to 0) in a 28-day period as assessed at the 12-week timepoint (week 9 to 12). The primary efficacy endpoint for the trial is the mean change in mean monthly migraine days (MMD) from baseline to the 12-week timepoint. An automated algorithm based on ICHD-3 criteria verifies for each day whether criteria for headache and migraine are met [24–26]. Secondary efficacy endpoints are defined as the mean change from baseline to the 12-week timepoint and include the number of monthly headache days (MHD), migraine attacks, and the amount of 50% responders, defined as patients who had ≥ 50% reduction in the number of migraine days. Secondary safety endpoints are the occurrence of adverse events and serious adverse events. Finally, exploratory endpoints are defined as the mean change from baseline in a 28-day period as assessed at the 12-week timepoint (weeks 9–12). The prespecified exploratory endpoints for this trial are as follows: (1) the amount of 75% responders, defined as patients who had ≥ 75% reduction in the number of migraine days; (2) the amount of complete responders, defined as patients who had 100% reduction in the number of migraine days; (3) total migraine hours; (4) acute medication treatment days; (5) migraine severity; (6) migraine-related symptom severity; (7) number needed to treat (NNT); (8) number needed to harm (NNH); (9) Migraine-Specific Quality of life questionnaire (MSQ); (10) Headache Impact Test (HIT-6); (11) Patient Global Impressions scales (PGI-S and PGI-I) and; (12) Perceived Stress Scale (PSS) (Fig. 2).

### Characteristics of participants

Female participants aged 18 or older with a diagnosis of menstrual migraine, who do not use hormonal supplementation and have had no changes to their prophylactic medication for at least 2 months, are eligible for this study (Tables 1 and 2). If participants are already using hormonal contraceptives, they should be willing to undergo a washout period of two consecutive months. Subjects will be recruited from our previous WHAT! diary cohort (P18.181), the Headache clinic at the Leiden Headache Center (LHC), and through national advertising and our dedicated websites (whatstudy.nl/en/ and hoofdpijnonderzoek.nl/en/). At our Headache Center, we employ electronic tools in the screening process for our patients. This approach is particularly important as we have previously demonstrated the limited accuracy of self-reported menstrual migraine diagnosis [25]. Recognizing the necessity of a prospective headache E-diary for a reliable menstrual migraine diagnosis, we mandate that all patients participating in research studies or receiving clinical care at the Leiden Headache Center complete these electronic diaries.

**Table 1:** Patient selection criteria

*Inclusion criteria*
• Female sex
• Age ≥ 18 years
• Menstrually related migraine or pure menstrual migraine (ICHD-3 criteria [25, 27])
• Demonstrated at least 80% compliance with the E-diary during the baseline period
• No or stable for at least 2 months on prophylactic medication
*Exclusion criteria*
• Migraine with aura with ≥ 50% of their migraine attacks accompanied by auras
• Chronic migraine (ICHD-3 criteria [27])
• Medication-overuse headache (ICHD-3 criteria [27])
• Women who are breastfeeding, pregnant, or planning to become pregnant
• Hormonal contraceptive use and not willing to undergo a washout period (stop for two consecutive months)
• Vitamin E use at start of the study
• Use of other sex hormone-containing treatments
• Increased risk of venous thromboembolism (VTE): history of VTE or thrombophlebitis, hereditary predisposition for VTE (APC resistance, protein C or S deficiency, antithrombin deficiency), VTE in first-degree family member at young age, long-term immobilization
• Increased risk of arterial thromboembolism (ATE): history of ATE, hereditary predisposition for ATE (hyperhomocysteinemia, antiphospholipid antibodies), ATE in first-degree family member at young age, diabetes mellitus, total cholesterol ≥ 6.5
• Other contraindication for combined oral contraceptives: liver malignancy, schistosomiasis, HIV/aids, use of immunosuppressives, tuberculosis, sex-hormone-dependent malignancies (breast, endometrial or ovary carcinomas), pancreatitis, vaginal bleeding with unknown cause, other diseases that can influence vessels (malignancies, heart valve disorders, atrial fibrillation, SLE, hemolytic uremic syndrome, chronic inflammatory bowel disease, sickle cell disease)
• Contraindication for vitamin E: vitamin K deficiency
• Hypersensitivity for any of the compounds in the combined oral contraceptive pill or vitamin E
• Spontaneous postmenopausal status (menstrual bleedings have ceased for 12 consecutive months)
• Iatrogenic postmenopausal status
• Inability to complete the E-diary in an accurate manner
• Any serious illness that can compromise study participation

**Table 2:** Trial registration data

Data category	Information
1. Primary registry and trial identifying number	ClinicalTrials.gov NCT04007874
2. Date of registration in primary registry	28 June, 2019
3. Secondary identifying numbers	2018–004096-12 (EudraCT)
4, Source(s) of monetary or material support	Netherlands Organization for Health Research (849200007) and the Dutch Brain Foundation (HA2017.01.05)
5. Primary sponsor	Leiden University Medical Center, Leiden, The Netherlands
6. Secondary sponsor(s)	Erasmus Medical Center, Rotterdam, The Netherlands
7. Contact for public queries	B.W.H.van_der_Arend@lumc.nl
8. Contact for scientific queries	B.W.H.van_der_Arend@lumc.nl
9. Public title	Oral Contraceptive Pill Compared With Vitamin E in Women With Migraine (WHATT)
10. Scientific title	Open-label Randomized Controlled Trial for the Effects of Continuous Ethinylestradiol/Levonorgestrel (30/150 μg/Day) Compared With Vitamin E (400 IU/Day) in the Treatment of Menstrually-related Migraine
11. Countries of recruitment	Netherlands
12. Health condition(s) or problem(s) studied	• Migraine • Migraine; menstrual
13. Intervention(s)	Active comparator: *ethinylestradiol/levonorgestrel* • *Ethinylestradiol/levonorgestrel 30/150 μg oral tablets once daily without a stop week for 3 months* • *Other names:* ◦ *Microgynon 30* ◦ *RVG 08204*
	Placebo comparator: *vitamin E* • *Vitamin E 400 IU oral capsules once daily for 3 months*
14. Key inclusion and exclusion criteria	Inclusion criteria: • Female • Menstrual migraine • Demonstrated at least 80% compliance with E-diary during baseline period • No or stable for at least two months on prophylactic medication
	Exclusion criteria: • Smoking • Migraine with aura • Chronic migraine with 15 or more headache days per month/with 8 or more migraine days per month • Medication-overuse headache (ICHD-3 criteria) • Women who are breastfeeding, pregnant, or planning to become pregnant • Oral contraceptive use and not willing to undergo washout period (stop for two consecutive months) • Vitamin E use at start of the study • Use of other sex hormone containing treatments • Increased risk of VTE: history of VTE or thrombophlebitis, hereditary predisposition for VTE (APC resistance, protein C or S deficiency, antithrombin deficiency), VTE in first-degree family member at young age, long-term immobilization • Increased risk of ATE: history of ATE, hereditary predisposition for ATE (hyperhomocysteinemia, antiphospholipid antibodies), ATE in first-degree family member at young age, diabetes mellitus, total cholesterol ≥ 6.5 • Other contraindication for oral contraceptives: liver malignancy, schistosomiasis, HIV/aids, use of immunosuppressives, tuberculosis, sex-hormone-dependent malignancies (breast, endometrial or ovary carcinomas), pancreatitis, vaginal bleeding with unknown cause, other diseases that can influence vessels (malignancies, heart valve disorders, atrial fibrillation, SLE, hemolytic uremic syndrome, chronic inflammatory bowel disease, sickle cell disease) • Contraindication for vitamin E: vitamin K deficiency • Hypersensitivity for any of the compounds in oral contraceptive or vitamin E • Spontaneous postmenopausal status (menstrual bleedings have ceased for 12 consecutive months) • Iatrogenic postmenopausal status • Inability to complete the electronic diary in an accurate manner • Any serious illness that can compromise study participation
15. Study type	Interventional
	Allocation: randomized intervention model. Parallel assignment masking: none (open-label)
	Primary purpose: prevention
	Phase III
16. Date of first enrolment	June 2019
17. Target sample size	180
18. Recruitment status	Recruiting
19. Primary outcome(s)	• Number of migraine days [time frame: from baseline to the last 4 weeks of treatment (weeks 9–12)] ◦ Change in monthly migraine days
20. Key secondary outcomes	• Number of headache days [time frame: from baseline to the last 4 weeks of treatment (weeks 9–12)] ◦ Change in monthly headache days • Number of migraine attacks [time frame: from baseline to the last 4 weeks of treatment (weeks 9–12)] ◦ Change in monthly migraine attacks • Number of probable migraine attacks [time frame: from baseline to the last 4 weeks of treatment (weeks 9–12)] ◦ Change in monthly probable migraine attacks • Number of 50% responders [time frame: from baseline to the last 4 weeks of treatment (weeks 9–12)] ◦ Patients who had ≥ 50% reduction in the number of migraine days • (Serious) adverse events [time frame: up to 3 months] ◦ Occurrence of adverse events and serious adverse events
21. Ethics review	Ethical approval was assigned by the CCMO of the Netherlands (NL67994.058.19). All participating patients will provide written informed consent
22. Completion date	2024–12 (Final data collection date for primary outcome measure)
23. Summary results	Not completed yet
24. IPD sharing statement	Plan to Share IPD: no

### Ethical considerations

The study is performed in accordance with the declaration of Helsinki Ethical Principles and Good Clinical Practices. The study design has been approved by the local and national ethics committees of the Netherlands (NL67994.058.19). In case of a protocol modification, the investigators, ethics committees, and clinical trial registries will be informed trough written communication. Informed consent is obtained from all participants before inclusion. No financial rewards will be granted to participants. Travel expenses will be compensated. A data monitoring committee (DMC) is established to oversee the safety and integrity of the study. This committee is an independent committee, consisting of an epidemiologist and a statistician. Both members have no conflict of interest with the principal investigator of the study. They are tasked with interim analyses on safety data and can advise on early termination of the trial. The advice of the DSMB will only be sent to the principal investigator of the study. Should the principal investigator decide not to fully implement the advice of the DSMB, the principal investigator will send the advice to the reviewing ethical committee of the Netherlands, including a note to substantiate why (part of) the advice of the DSMB will not be followed. Auditing is conducted twice a year at the LUMC by an internal pool of monitors. The auditing process is independent of both the investigators and the sponsor.

### Study procedures

Subjects with proven menstrual migraine according to their headache electronic diary (E-diary) data or subjects who have indicated an interest via our web form will receive an information form, an informed consent form, and a response leaflet as an invitation to participate in this trial [25]. If there is no response within 2 weeks, a telephone call will be made by a researcher to provide further information. Participants will visit the LHC to determine eligibility. For this purpose, initial history (including cardiovascular risk factors and comorbidity) and neurological examination (including also blood pressure measurement) will be performed. A blood sample will also be taken to determine hormone and cholesterol status. If participants give permission, 40 ml extra blood will be collected and stored for biobanking purposes in the LUMC Biobank Headache (neurological disorders). The regulations of the LUMC Biobank Headache Collection protocol will be applicable to this biobank collection, registered under BB23.003 (the Netherlands).

After the first visit to the LHC, participants will start with a baseline period of 1 month. During the baseline period, participants start or continue filling out a daily headache E-diary. After completion of this month, remaining exclusion criteria will be checked (chronic migraine and medication-overuse headache). If a woman is found eligible, she will be randomized for either the combined oral contraceptive pill or vitamin E using Castor EDC [28]. Before the start of treatment, all participants will take a urine pregnancy test and complete questionnaires (15 min). During the baseline visit, participants will receive an invitation to fill out an online expectations questionnaire, the Stanford Expectations of Treatment Scale (SETS). This questionnaire will be used to help assess expectancy effects of both interventions. For participants whom already registered 1 month of E-diary data prior to the inclusion visit, additional baseline measurements are not needed. The questionnaires filled out during this period serve as baseline measurements for these participants.

The baseline period is followed by the intervention period of three months. Participants will start with either ethinylestradiol/levonorgestrel 30/150 μg or vitamin E 400 IU once daily. We will actively implement strategies to enhance participant retention in this study. This proactive approach involves monthly contact with patients throughout the treatment period. Regular communication aims to strengthen the engagement and commitment of participants to the study, thereby minimizing the likelihood of attrition. Participants on oral contraceptives will be strongly encouraged to take the medication without a stop week if mild spotting or break through bleeding occurs. In case of long lasting or unacceptable break through bleedings, women are allowed to skip the combined oral contraceptive pill for 7 days, during which they will be prescribed ethinylestradiol 25 μg once daily for 7 days. Participants should continue other prescribed prophylactic treatments and are allowed to take their acute treatment in case of a migraine attack.

Participants will be contacted twice (*t* = 1 and *t* = 2) to evaluate (serious) adverse events ((S)AEs), which will be asked using open-ended questions. Follow-up visit 3 (*t* = 3) includes questionnaires (15 min) and evaluation of therapy (Fig. 1). If necessary, regular clinical follow-up visits will be planned.

### Data storage

All data gathered during this study will be stored in compliance with the European Union General Data Protection Regulation (EU GDPR). Data collection will be conducted by authorized investigators using two-factor authenticated, password-protected web-based interfaces, ensuring secure access. To further safeguard participant privacy, all data will be assigned a unique four-digit study code that cannot be linked back to the participant’s identity. Participants can withdraw their consent for the current use of their data, as well as for use in future research, at any time without consequences.

### Sample size calculation

To achieve the primary objective of the study, at least 168 participants are estimated to be needed for random allocation into the two treatment arms (84 per group). The study is designed to have 90% power and a type 1 error of 0.05 for the detection of a between-group difference in MMD of 2 days. Based on previous studies on preventive therapy, we expect a standard deviation (SD) of 4.0 days for the MMD.

$$n(\mathrm{number}\;\mathrm{needed}\;\mathrm{per}\;\mathrm{group})=\left(1.96+1.28\right)^2\times\left(\left(2\times\text{SD}^2\right)/\left(\text{diff}^2\right)\right)$$

$$n = 10.5 \times \left(32/4\right) = 84$$

Considering potential drop-out, we aim to include *n* = 90 participants per treatment group (*n* = 180 in total) (Fig. 1).

### Outcome measurements

An intention-to-treat approach (all participants who were randomly assigned and received at least one dose of study drug) will be used for the primary analysis. In addition, an as-treated analysis will be performed with only those participants who used study medication daily during three months (without a stop week and estrogen supplementation). A *p*-value less than 0.05 will be considered significant. All statistical analysis will be performed using R.

The primary endpoint is change in MMD from baseline to the last 4 weeks of treatment (weeks 9–12). Group differences (ethinylestradiol/levonorgestrel 30/150 μg versus vitamin E 400 IU) will be analyzed using a mixed-effects model of repeated measures with patients’ baseline value and treatment as fixed effects and patients as random effects. Secondary continuous efficacy endpoints will be analyzed using a linear mixed-effects model including appropriate terms and covariates. The adjusted mean change from baseline for each treatment group, associated 95% confidence intervals, and *p*-values will be reported. Dichotomous endpoints derived from corresponding continuous endpoints will be analyzed with stratified Cochran-Mantel–Haenszel tests. For dichotomous efficacy endpoints, adjusted odds ratios for ethinylestradiol/levonorgestrel compared with vitamin E, associated 95% confidence intervals, and *p*-values will be reported. The primary end point and continuous secondary end points will be analyzed without any imputation of missing data. Sensitivity analyses will be conducted with imputation under missing-at-random or missing-not-at-random assumptions. The data analyst will be blinded.

Safety analysis will include all participants who received at least one dose of study drug. AEs and SAEs will be coded and summarized by Medical Dictionary for Regulatory Activities. All adverse events will be documented.

## Discussion

This article presents the rationale and design of an ongoing randomized controlled trial that aims to investigate the preventive effect of continuous daily use of ethinylestradiol/levonorgestrel (30/150 μg/day) compared with vitamin E (400 IU/day) in women with menstrual migraine. Our trial is designed to provide evidence for the effectiveness of the combined oral contraceptive pill as a preventive treatment for menstrual migraine. Vitamin E is being used as a placebo control group in this trial. The choice of vitamin E as a placebo was based on its limited efficacy in the prevention of menstrual migraine attacks and its good tolerability profile [21]. This will allow for a fair comparison between the two treatment groups while minimizing the risk of bias. To correct for the unblinded settingof the study, the Stanford Expectations of Treatment Scale will be used [29]. This scale is designed to assess the expectations of participants regarding the effectiveness of the treatment they receive. By using this scale, we can measure and adjust for any potential differences in expectations between the two treatment groups, which could influence the outcomes of the trial.

Menstrual migraine affects an important proportion of women of reproductive age, as it concerns two thirds of all women with migraine [25]. Despite the availability of prophylactic medications, many women still experience frequent attacks, particularly during the menstrual window [30]. These perimenstrual attacks are often longer of duration with increased risk of recurrence, more severe, and less responsive to acute medication [27]. Previous studies have suggested that the use of combined oral contraceptives, such as ethinylestradiol/levonorgestrel, can reduce the frequency and severity of perimenstrual migraine attacks. However, the current evidence is limited and conflicting, and the optimal dosing regimen and duration of treatment remain unclear. Therefore, this large randomized controlled trial is an imperative necessity to determine the efficacy of the continuous use of ethinylestradiol/levonorgestrel in women with menstrual migraine.

## Conclusion

The results of the WHAT! trial will have important implications for the clinical management of women with menstrual migraine and can be implemented in future guidelines and recommendations regarding the use of combined oral contraceptive pills as a preventive treatment.

## Trial status

Recruiting started on 10t September 2019. The current protocol is version 5.0 of 01 August 2022. Currently (12t October 2023), we included 106 patients. Patient recruitment is estimated to be completed around December 2024.

## Data Availability

The data that support the findings of this study are available on reasonable request from the corresponding author. Trial results will be communicated to participants, healthcare professionals, the public, and other relevant groups trough publication.

## Abbreviations

WHAT: Women, Hormones, Attacks and Treatment
COC: Combined oral contraceptives
CHC: Combined hormonal contraception
HFI: Hormone-free interval
MMD: Monthly migraine days
MHD: Monthly headache days
RCT: Randomized controlled trial
LHC: Leiden Headache Center
E-diary: Electronic diary
SETS: Stanford Expectations of Treatment Scale
SAE: Serious adverse event
AE: Adverse event
SD: Standard deviation
